# Arabidopsis SHR and SCR transcription factors and AUX1 auxin influx carrier control the switch between adventitious rooting and xylogenesis *in planta* and in *in vitro* cultured thin cell layers

**DOI:** 10.1093/aob/mcu258

**Published:** 2015-01-22

**Authors:** F. Della Rovere, L. Fattorini, S. D’Angeli, A. Veloccia, S. Del Duca, G. Cai, G. Falasca, M. M. Altamura

**Affiliations:** ^1^Department of Environmental Biology, Sapienza University of Rome, Italy, ^2^Department of Biological, Geological and Environmental Sciences, University of Bologna, Italy and ^3^Department of Life Sciences, University of Siena, Italy

**Keywords:** Adventitious rooting, *Arabidopsis thaliana*, auxin influx carriers, AUX1, LAX3, SCR, SHR, thin cell layers, xylogenesis

## Abstract

**Background and Aims** Adventitious roots (ARs) are essential for vegetative propagation. The *Arabidopsis thaliana* transcription factors SHORT ROOT (SHR) and SCARECROW (SCR) affect primary/lateral root development, but their involvement in AR formation is uncertain. LAX3 and AUX1 auxin influx carriers contribute to primary/lateral root development. *LAX3* expression is regulated by SHR, and LAX3 contributes to AR tip auxin maximum. In contrast, AUX1 involvement in AR development is unknown. Xylogenesis is induced by auxin plus cytokinin as is AR formation, but the genes involved are largely unknown. Stem thin cell layers (TCLs) form ARs and undergo xylogenesis under the same auxin plus cytokinin input. The aim of this research was to investigate SHR, SCR, AUX1 and LAX3 involvement in AR formation and xylogenesis in intact hypocotyls and stem TCLs in arabidopsis.

**Methods** Hypocotyls of *scr-1*, *shr-1*, *lax3*, *aux1-21* and *lax3/aux1-21 Arabidopsis thaliana* null mutant seedlings grown with or without auxin plus cytokinin were examined histologically, as were stem TCLs cultured with auxin plus cytokinin. *SCR* and *AUX1* expression was monitored using *pSCR::GFP* and *AUX1::GUS* lines*,* and *LAX3* expression and auxin localization during xylogenesis were monitored by using *LAX3::GUS* and *DR5::GUS* lines.

**Key Results** AR formation was inhibited in all mutants, except *lax3*. *SCR* was expressed in pericycle anticlinally derived AR-forming cells of intact hypocotyls, and in cell clumps forming AR meristemoids of TCLs. The apex was anomalous in *shr* and *scr* ARs. In all mutant hypocotyls, the pericycle divided periclinally to produce xylogenesis. Xylary element maturation was favoured by auxin plus cytokinin in *shr* and *aux1-21*. Xylogenesis was enhanced in TCLs, and in *aux1-21* and *shr* in particular. *AUX1* was expressed before *LAX3*, i.e. in the early derivatives leading to either ARs or xylogenesis.

**Conclusions** AR formation and xylogenesis are developmental programmes that are inversely related, but they involve fine-tuning by the same proteins, namely SHR, SCR and AUX1. Pericycle activity is central for the equilibrium between xylary development and AR formation in the hypocotyl, with a role for AUX1 in switching between, and balancing of, the two developmental programmes.

## INTRODUCTION

Adventitious roots (ARs) may contribute together with the primary root (PR) and lateral roots (LRs) to form the root apparatus and are essential for vegetative propagation. The transcription factors SHORT ROOT (SHR) and SCARECROW (SCR), belonging to the GRAS family, are key regulators of PR stem cell definition/maintenance and radial patterning (Di Laurenzio *et al.*, 1996; Helariutta *et al.*, 2000; Sabatini *et al.*, 2003). *SHR* is expressed in the stele, and SHR moves into the adjacent layer to control *SCR* transcription and endodermis specification (Koizumi *et al.*, 2012). In contrast, *SCR* is expressed in the cortex/endodermis initial cell and the endodermis, its protein binds to its own promoter in the presence of SHR (Cui *et al.*, 2007), and they jointly regulate quiescent centre (QC) markers (e.g. WOX5; Sarkar *et al.*, 2007) and microRNAs (i.e. microRNA 165/6) involved in PR vascular differentiation (Carlsbecker *et al.*, 2010).

Cytokinin is involved with auxin in controlling PR apical growth and vascular patterning, with SHR regulating cytokinin homeostasis in the xylem-associated pericycle (Cui *et al.*, 2011; Zhang *et al.*, 2013). Auxin and cytokinin are also involved in LR formation (Benková *et al.*, 2003; Bielach *et al.*, 2012), as are SHR (Lucas *et al.*, 2011) and SCR (Malamy and Benfey, 1997), and *SCR* is auxin induced (Moubayidin *et al.*, 2013).

AUXIN RESISTANT1 (AUX1) and LIKE AUXIN RESISTANT3 (LAX3) are auxin influx carriers of the same family. In arabidopsis they are required for QC organization in the embryonic radicle (Ugartechea-Chirino *et al.*, 2010), are PR vascular markers also involved in different phases of the LR formation process (Swarup *et al.*, 2008), and LAX3 is required for defining the auxin maximum and maintenance in the AR tip *in planta* (i.e. in ARs from intact hypocotyls) and in cultured thin cell layers (TCLs) (Della Rovere *et al.*, 2013). *LAX3* expression is regulated by SHR (Sozzani *et al*., 2010), but information about SHR involvement in AR formation is still contradictory (Scheres *et al.*, 1995; Lucas *et al.*, 2011). Stem TCLs are explants devoid of vasculature, i.e. the site of *SHR* expression *in planta* (Helariutta *et al.*, 2000), but are capable of AR initiation by the endodermis (Falasca *et al.*, 2004), i.e. the tissue lacking in the stems of *shr* and *scr* mutants (Fukaki *et al.*, 1998; Wysocka-Diller *et al.*, 2000). Moreover, AR formation in TCLs needs exogenous auxin [indole-3-butyric acid (IBA) (10 µm)] combined with cytokinin [kinetin (Kin) (0·1 µm)]. A role for AUX1 in controlling organ identity by mediating auxin–cytokinin interaction has been suggested in arabidopsis calli (Kakani *et al.*, 2009), as a role for LAX3 in auxin/ cytokinin distribution during AR development (Della Rovere *et al.*, 2013). However, whether these auxin influx carriers are involved with SHR and SCR in the control of AR formation from TCLs has never been investigated.

Both *in planta* and in cultured explants, xylogenesis consists of ectopic formation of tracheary-like cells, and in many species is an auxin plus cytokinin-mediated programme (Fukuda, 1997). Xylogenesis has led to important discoveries in the understanding of xylem formation *in planta*, and genes in common between the xylary processes *in planta* and *in vitro* have been found (Miyashima *et al.*, 2013). Arabidopsis TCLs show xylogenesis in addition to AR formation under the same IBA + Kin input (Falasca *et al.*, 2004), and the intact hypocotyl is induced to produce xylogenesis under specific auxin types/treatments (Falasca and Altamura, 2003). The possibility that AR formation and xylogenesis are under the control of common genes needs investigation.

The aim of the present research was to determine SHR and SCR involvement, in relation to the activities of AUX1 and LAX3, during AR formation and xylogenesis, in intact hypocotyls in the absence/presence of exogenous IBA and Kin, and in IBA + Kin-cultured TCLs, by investigating gene expression patterns and single/double mutant responses.

The results showed that AR formation and xylogenesis are inversely related, with *SHR*, *SCR* and *AUX1* controlling the fine-tuning between the morphogenic programmes. The role of these transcription factors in the initiation of both processes, and of AUX1 in their switching, is discussed.

## MATERIALS AND METHODS

### Plant material and growth conditions

*Arabidopsis thaliana* seeds of the homozygous *scr-1* and *shr-1* [Wassilewskija (Ws) wild type (wt)] and *lax3*, *aux1-21* and *lax3/aux1-21* [Columbia (Col) wt] null mutants, and of *pSCR::GFP* (Ws background), *AUX1::GUS*, *LAX3::GUS* and *DR5::GUS* lines (Col background) were stratified and sterilized according to Della Rovere and co-workers (2013). These seeds (15–20 per plate, 20 plates per genotype, 12 × 12 cm each) were either sown on hormone-free (HF) medium and with IBA (10 µm) and Kin (0·1 µm) (IBA + Kin), or on a commercial soil, for seedling growth and plant production, respectively, according to Della Rovere *et al.* (2013). For seedling growth, the plates were placed in a vertical position at 22 ± 2 °C under continuous darkness for 14 d, after exposure to white light for 6 h (Takahashi *et al*., 2003).

### TCL culture

Superficial TCLs, about 0·5 × 8 mm, 6–7 layers thick, were excised from the inflorescence–stem internodes of thirty 35-day-old plants of *pSCR::GFP*, *AUX1::GUS*, *LAX3::GUS*, *DR5::GUS*, *shr-1*, *scr-1*, *lax3*, *aux1-21*, *lax3/aux1-21* and the corresponding wt. One hundred TCLs per genotype were cultured up to day 20 under IBA + Kin. Explants were examined under a Leica MZ8 stereomicroscope at the end of the culture period, and the response was evaluated as the mean number of ARs (± s.e.) per rooting explant.

### Histological analysis under light and fluorescence/confocal microscopy

Thirty randomly selected seedlings per genotype and treatment were cleared, mounted and observed with Nomarski optics, and *AUX1::GUS*, *LAX3::GUS* and *DR5::GUS* seedlings were processed for β-glucuronidase (GUS) staining before clearing. In all mutants, and their wt, the hypocotyl length was measured under a Leica MZ8 stereomicroscope before fixation, and the number of ARs was expressed as mean density cm^−1^ (± s.e.).

Green fluorescent protein (GFP) fluorescence in *pSCR::GFP* was observed either under the Leica DMRB microscope equipped with a double wavelength filter set (BP 490/20 and BP 575/30) with dichroic filters RKPs 505 and 600 and emission filters BPs 525/20 and 635/40, or under confocal microscopy using a ×63 oil-immersion lens on a Leica TCS-SP5 confocal microscope supplied with the Leica application suite advanced fluorescence (LAS AF Lite) software (Leica Microsystems). Propidium iodide staining and detection were according to Della Rovere *et al.* (2013).

Basal stem internodes of *scr-1*, *shr-1* and wt plants were dehydrated, embedded, cross-sectioned (4 µm thickness) and stained with toluidine blue, according to Della Rovere and co-workers (2013).

Ten TCLs per genotype were harvested periodically for histology in bright field, as were 30 seedlings per genotype and treatment. Explants and seedlings were fixed, dehydrated and embedded, as above, longitudinally sectioned (4 µm thickness) and stained according to Della Rovere *et al.* (2013). Explants of the GUS marker lines were sectioned at 12 µm.

The histological image acquisition, the computerized analysis and numbering of the AR stages were according to Della Rovere and co-workers (2013).

### Xylogenesis counting

Twenty TCLs per mutant, and the corresponding wt, were fixed, dehydrated, sectioned and stained as above, and vascular strands, nodules and single lignified cells were counted under light microscopy at 70 µm intervals [interval established on the basis of the mean dimensions of the xylary cells, i.e. 42·2 (±3·6) × 11·6 (±1·7) × 10 (±1·3) µm]. The presence of lignin thickenings in the cell walls was verified by detecting lignin autofluorescence under a Zeiss Axiolab epifluorescence microscope, equipped with a 50 W HBO mercury lamp, using the BP 365, FT 395 and LP 397 filter set.

### Statistical analysis

Data were expressed as means (± s.e.). A normality test was applied before analysis of variance (ANOVA; GraphPad Instat 3). One-way/two-way ANOVA (*P* < 0·05) was used to compare the effects of genotypes, and genotypes and treatments, respectively, followed by Tukey’s post-test (GraphPad Prism 6.0). Experiments were repeated three times in two consecutive years, with similar results (data of the second year are shown).

## RESULTS

### The hypocotyl pericycle shows periclinal divisions leading to ectopic metaxylem formation in all the null mutants

**Fig. 1.**
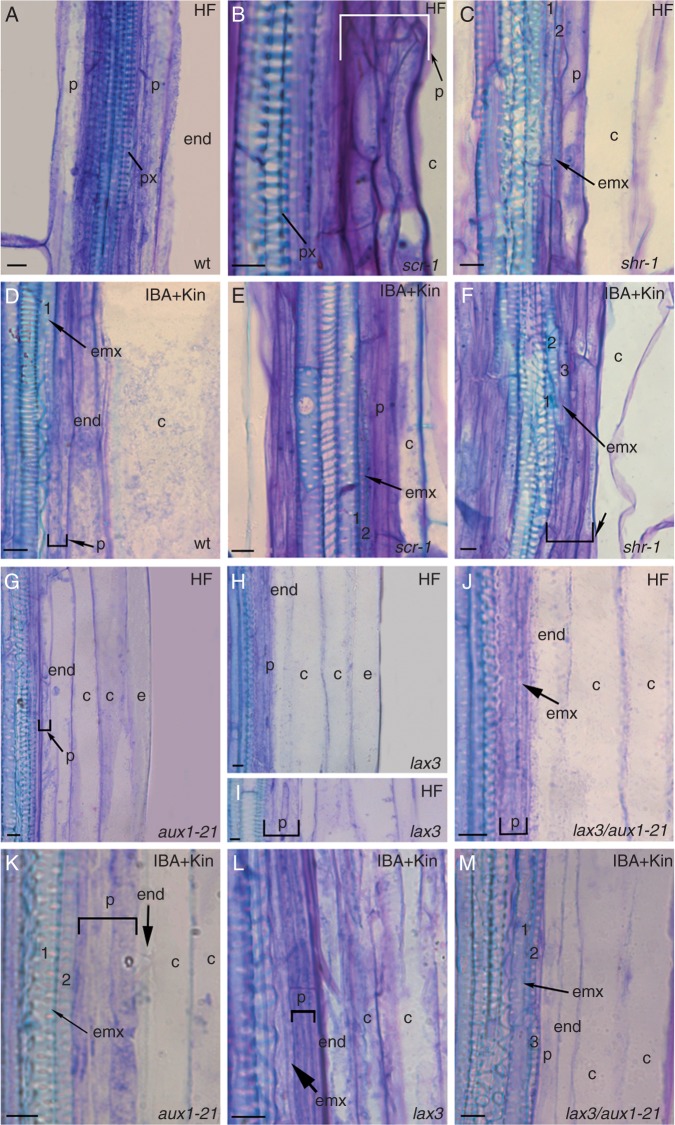
Ectopic metaxylem formation in pericycle proliferation at the hypocotyl–PR junction of 14-day-old wt (Ws, A, D), *scr-1* (B, E), *shr-1* (C, F), *aux1-21* (G, K), *lax3* (H, I, L) and *lax3/aux1-21* (J, M) seedlings grown under continuous darkness without hormones (HF) and with IBA + Kin. Longitudinal sections stained with toluidine blue. Numbers mark immature/mature ectopic metaxylem elements, and square brackets indicate the pericycle periclinal proliferation. Exarch metaxylem within the hypocotyl stele of *scr-1* (B) and *shr-1* (C) in comparison with the exarch protoxylem of the wt (A), *aux1-21* (G), *lax3* (H, I) and *lax3/aux1-21* (J) is shown. c, cortical parenchyma, e, epidermis, end, endodermis, emx, ectopic-metaxylem, p, pericycle, px, protoxylem. Scale bars = 10 µm.

The hypocotyl of *shr-1* and *scr-1* HF-grown seedlings exhibited two irregular cortical layers, no endodermis, exarch metaxylem (i.e. metaxylem in a peripheral position in the stele); and endarch protoxylem (i.e. protoxylem located internally to the metaxylem), in contrast to the regular structure of the wt (Fig. 1A–C). However, as in the wt, protoxylem was formed by tracheary elements with annular or helical thickenings, and metaxylem by pitted elements (Fig. 1A–C). At the *shr-1* and *scr-1* hypocotyl–PR junction, the pericycle divided periclinally, generating a multilayered bundle of meristematic cells. However, in *shr-1*, immature ectopic metaxylem (i.e. xylem elements not yet lignified but with pitted secondary thickenings forming) appeared in the bundle under HF conditions, and mature elements (i.e. dead pitted elements with lignified cell walls) appeared under IBA + Kin treatment (Fig. 1C, F). Immature ectopic metaxylem was observed in *scr-1*, and occasionally in the wt, under IBA + Kin treatment only (Fig. 1D, E).

The hypocotyl of *aux1-21*, *lax3* and *lax3/aux1-21* HF seedlings showed the same radial pattern as the wt, with the addition of a multilayered bundle of periclinally divided pericycle cells at its junction with the PR (Fig. 1G–J), where ectopic immature metaxylem occurred in the double mutant (Fig. 1J). Under IBA + Kin treatment, ectopic immature metaxylem appeared in all mutants (Fig. 1K–M), and occasionally in the wt, but mature elements appeared only in *aux1-21* and *lax3/aux1-21* (Fig. 1K, M).

### SCR and AUX1 are expressed from the AR-forming divisions in the hypocotyl pericycle

**Fig. 2.**
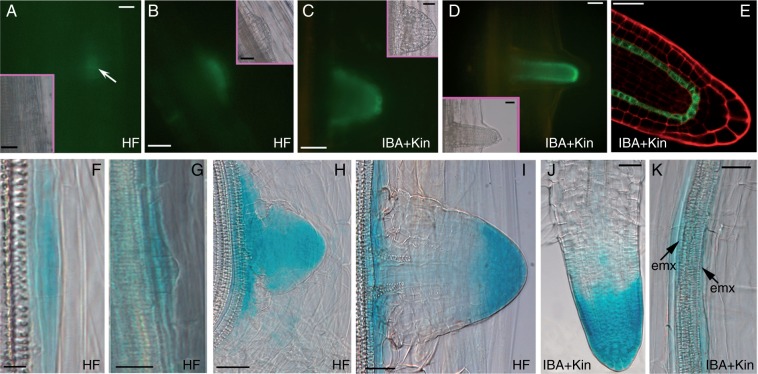
*SCR* (A–E), *AUX1* (F, H–K) and *LAX3* (G) expression in the AR-forming hypocotyl of 14-day-old *pSCR::GFP*, *AUX1::GUS* and *LAX3::GUS* seedlings grown under continuous darkness, either under HF conditions (A, B, F–I) or with IBA + Kin (C–E, J, K). (A) Appearance of *SCR* signal (arrow) at AR stage I and (B) signal presence at AR stage III. (C, D) *SCR* expression in the QC, endodermis/cortical initials and derivatives at AR stage VII (C) and in the ARP tip (D). (E) *SCR* expression in the QC, endodermis/cortical initials/derivatives and endodermis in the AR tip. (A–D, microscopic fluorescence pictures, with corresponding bright-field images in the insets; E confocal microscopy). (F, G) *AUX1* expression at stage I (F) and *LAX3* expression at stage II (G). (H, I) Uniform *AUX1* expression at stage VII (H) and expression limited to the tip and base in the ARP (I). (J) Detail of a mature AR expressing *AUX1* in all tip tissues. (K) *AUX1* expression in IBA + Kin periclinally divided pericycle and in the ectopic metaxylem (emx, arrows). Scale bars = 10 µm (A, F and inset in A) and 30 µm (B–E, G–K and insets in B–D).

*SCR* signal, monitored in *SCR::GFP* seedlings grown with or without exogenous hormones, appeared in the first anticlinally formed derivatives of the hypocotyl pericycle (AR stage I, Fig. 2A, arrow), increased at AR stage II/III (Fig. 2B) and was increased further at stage VII, localizing in the QC, endodermis/cortical initials and derived cells (Fig. 2C). *SCR* expression extended from the latter cells to the differentiating endodermis in AR primordia (ARPs) and mature ARs (Fig. 2D, E).

Endogenous auxin accumulated in the pericycle of the *DR5::GUS* seedlings at the hypocotyl–PR junction before any morphogenic event, increased at AR stage I and continued to be present at further AR stages, progressively localizing at the AR tip and the AR vascular connection with the hypocotyl. Auxin signal increased under IBA + Kin treatment, confirming previous results (Della Rovere *et al*., 2013).

In *AUX1::GUS* seedlings, under both HF and IBA + Kin conditions, *AUX1* was expressed in the hypocotyl pericycle AR stage I onwards(Fig. 2F), preceding the expression of *LAX3*, which initiated at AR stage II (Fig. 2G). *AUX1* continued to be uniformly expressed up to stage VII (Fig. 2H). In protruding ARPs and mature ARs, *AUX1*continued to be expressed over the whole tip, and in the procambium and differentiating protoxylem at the organ base (Fig. 2I, J). In contrast, *LAX3* expression was excluded from the AR tip, with the exception of the inner cap layer, but was present in all the AR differentiating/mature stele tissues (Della Rovere *et al*., 2013; and data not shown). In contrast to *LAX3*, *AUX1* expression did not increase under IBA + Kin treatment in comparison with HF conditions. Interestingly, occasional pericycle periclinally divided cells engaged in ectopic metaxylem formation showed *AUX1* expression (Fig. 2K, arrows).

### AR formation in intact hypocotyls is highly reduced in all mutants, except lax3

**Fig. 3.**
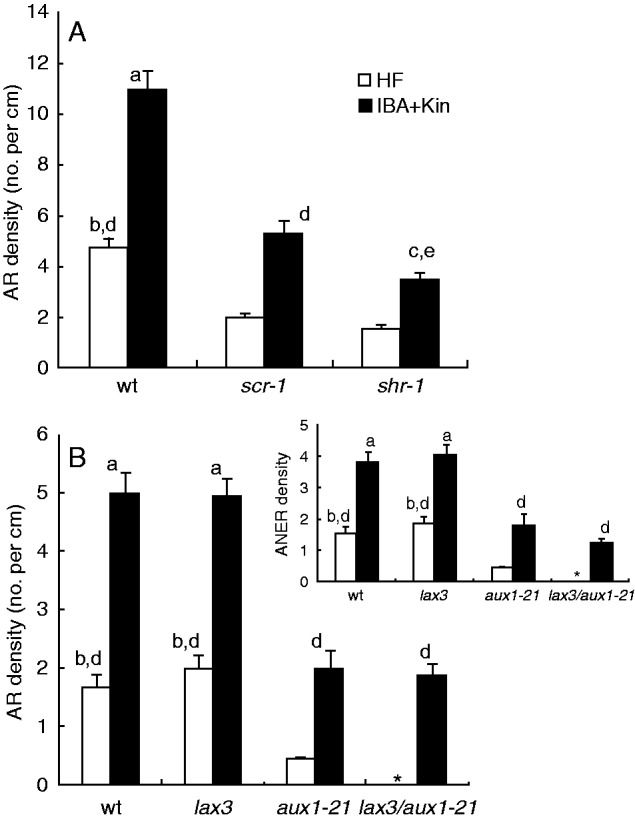
AR mean density (± s.e.) in the hypocotyl of 14-day-old wt (Ws), *scr-1* and *shr-1* (A), and wt (Col), *lax3*, *aux1-21* and *lax3/aux1-21* (B) seedlings grown under continuous darkness on HF and IBA + Kin. Emerged ARs and ARs only histologically detectable (ANERs) were counted together (A, B). The asterisk shows no AR response. ANER density alone is shown in the inset. ^a^*P* < 0·0001 with other genotypes within the IBA + Kin treatment, ^b^*P* < 0·0001 with other genotypes within the HF treatment. ^c^*P* < 0·05 with *scr-1* within the IBA + Kin treatment. ^d^*P* < 0·0001 within the same genotype. ^e^*P* < 0·05 within the same genotype. Columns with no letter/the same letters are not significantly different (two-way ANOVA followed by Tukey’s post-test). *n* = 30 (second replicate of the second year).

At day 14, independently of the treatment, the density of the emerged and non-emerged ARs on the hypocotyls was significantly lower than in the wt (Fig. 3A). The IBA + Kin treatment increased the AR density, significantly more in *scr-1* than in *shr-1* (Fig. 3A).

Macroscopic ARs were rare, and non-emerged ARs (ANERs) were prevalent on both HF-treated wt and *lax3* seedlings, without differences between the two (Fig. 3B and inset). In contrast, the AR density of HF-treated *aux1-21* seedlings was very low, and almost totally constituted by ANERs, whereas no AR stage occurred in the HF-treated *lax3/aux1-21* seedlings (Fig. 3B and inset). Under IBA + Kin treatment, in contrast to *lax3* and the wt, the *aux1-21* AR response remained almost totally constituted by ANERs, but the density increased in comparison with the HF treatment, as in *lax3* and the wt, remaining, however, less than the half that of the latter genotypes (Fig. 3B and inset). Moreover, the IBA + Kin treatment caused AR formation in the double mutant, with a response similar to *aux1-21* (Fig. 3B and inset).

### AR formation in intact hypocotyls is altered in all mutants, although at different levels

**Fig. 4.**
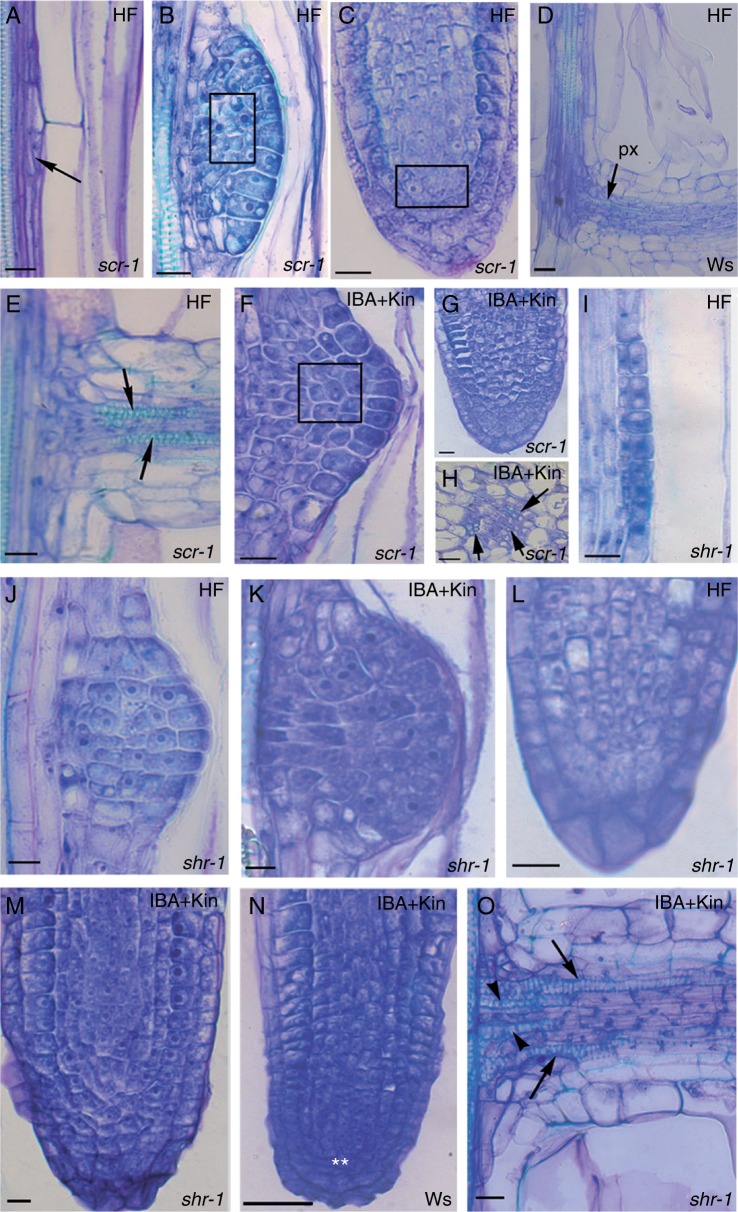
*scr-1* (A–C, E–H) and *shr-1* (I–M, O) AR formation at the hypocotyl–PR junction of 14-day-old seedlings grown under continuous darkness under either HF or IBA + Kin treatment, in comparison with the wt (D, N). (A–C) Anomalous divisions at stage I (A, arrow), and no QC definition in the region marked by a rectangle either at stage VII (B) or in the AR apex (C) of HF *scr-1-*seedlings. (D, E) Exarch protoxylem differentiation (px, arrows) at the base of *scr-1* ARs (E) and wt-ARs (D) under HF treatment. (F, G) Anomalous AR development in IBA + Kin *scr-1* seedlings from stage VII (F) to mature tip formation (G). The square in F shows the region where the QC is not defined. (H) Xylem overproduction (arrows) in the stele of *scr-1* ARs produced under IBA + Kin treatment. (I) Outer pericycle derivatives leading to AR stage II in HF *shr-1* seedlings. (J, K) *shr-1* stage VII lacking QC definition under HF (J) and IBA + Kin treatment (K). (L, M) *shr-1* mature AR tips with no QC under HF (L) and IBA + Kin (M) treatment, and with a cell enrichment in the latter. (N) Regular apical structure of an AR formed by a IBA + Kin-treated wt seedling. The QC cells are marked by the asterisks. (O) Vascular connection with the hypocotyl of an IBA + Kin-treated *shr-1* AR showing exarch protoxylem (arrows) and endarch metaxylem (arrowheads). Longitudinal (A–G and I–O) and transverse (H) sections stained with toluidine blue. Scale bars = 10 µm (G, I–M, O), 20 µm (A–C, E, F, H) and 30 µm (D, N).

At the hypocotyl–PR junction of HF-treated *scr-1* seedlings, the external derivatives of the periclinally divided pericycle bundle occasionally showed oblique/anticlinal divisions (Fig. 4A, arrow), followed by AR formation. In comparison with the wt (Della Rovere *et al.*, 2013), in the *scr-1* mutant, the QC was not defined either at stage VII or later, resulting in absence of the QC in the apex of the rare mature ARs (Fig. 4B, C). However, as in the wt, vascular elements with protoxylem-like thickenings were formed in the *scr-1* mutant at the point of connection of the AR with the hypocotyl vasculature, and differentiated into the AR exarch protoxylem (Fig. 4D, E, arrows). The HF-treated *scr-1* ARs were diarch, with the same wt xylem patterning, but lacking the endodermis. In the presence of IBA + Kin, the AR-forming divisions in the pericycle also occurred sporadically along the hypocotyl, but resulted in anomalous stage VII domes (Fig. 4F), as under HF treatment. However, the apex of the mature ARs was enriched in meristematic cells in comparison with HF treatment, even if there continued to be a lack of cells with QC morphology (Fig. 4G). The endodermis was absent in the IBA + Kin-treated *scr-1* ARs as was the case under HF treatment, but xylem formation was enhanced in the stele, frequently leading to triarch ARs (Fig. 4H). At the hypocotyl–PR junction of HF- and IBA + Kin-grown *shr-1* seedlings, AR-forming divisions occurred sporadically, but always in the outer layer of the periclinally divided pericycle bundle (Fig. 4I). In contrast to the wt, in *shr-1*, as for *scr-1*, QC definition did not occur either at stage VII or later (Fig. 4J, K), resulting in the QC being absent in mature ARs in both treatments (Fig. 4L, M; compare with C, G, N). However the apex of the ARs formed under IBA + Kin treatment was enriched in meristematic and root cap cells in comparison with the HF treatment (Fig. 4L, M), and the AR connection with the hypocotyl was enriched in vascular elements (Fig. 4O, arrows). In the AR primary structure, endodermis was absent, and protoxylem was exarch. Triarch ARs appeared under IBA + Kin treatment, as in *scr-1* under the same treatment.

**Fig. 5.**
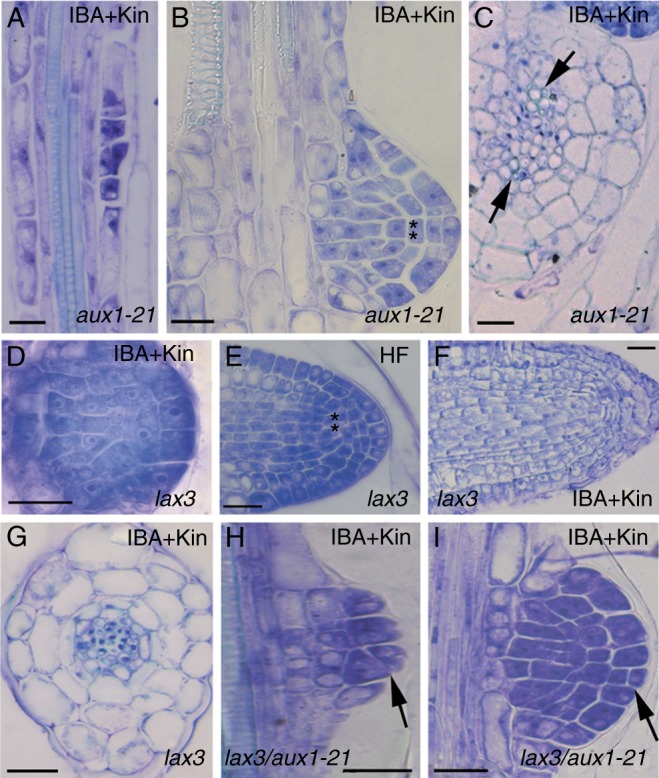
AR formation in *aux1-21* (A–C), *lax3* (D–G) and *lax3/aux1-21* (H, I) hypocotyl of 14-day-old seedlings grown under continuous darkness with either IBA + Kin or HF treatment. (A, B) First AR stages along an *aux1-21* hypocotyl (A) and more advanced stages, e.g. stage VII (B), at its junction with the PR under IBA + Kin treatment. The regular presence of the QC at stageVII is marked by asterisks. (C) Regular radial pattern but xylem overproduction within the stele (arrows) in mature ARs (IBA + Kin). (D, E) AR stage VII lacking QC definition, in IBA + Kin-treated *lax3* seedlings (D), and QC presence, marked by the asterisks, in the tip of a not yet protruded ARP of a *lax3* HF seedling (E). (F, G) Details of a mature AR of a IBA + Kin-treated *lax3* seedling showing an apex, regular in structure, but enriched in root cap cells (F), and primary structure definition with a regular radial pattern (G). (H, I) AR stages in IBA + Kin-treated *lax3/aux1-21* seedlings. Anomalous divisions at stage V (H) and in the root cap initials at stage VII (I) are shown by the arrows. Longitudinal (A, B, D–F, H, I) and transverse (C, G) sections stained with toluidine blue. Scale bars = 20 µm.

In both HF- and IBA + Kin-treated *aux1-21* seedlings initial AR stages (Fig. 5A) were more frequent than advanced AR stages. The rare *aux1-21* stage VII domes showed regular QC definition (Fig. 5B, asterisks), and developed into ARPs with regular apical structure. The rare protruding *aux1-21* ARs were located at the hypocotyl–PR junction, and showed a regular primary radial pattern, but with overproduction of xylem (Fig. 5C, arrows).

The first pericycle divisions in *lax3* seedlings were regularly anticlinal, under both HF and IBA + Kin treatment, and led to dome-shaped ARPs, with QC definition not occurring at stage VII (Fig. 5D), but instead occurring later (Fig. 5E, asterisks). There were numerous ARPs advanced in development, and they showed periclinal divisions in root cap initials leading to ARs with multilayered root caps (Fig. 5F). Under both treatments, in *lax3* ARs the definition of the primary radial pattern was regular (Fig. 5G), as was the acropetal differentiation and the position of the protoxylem.

At the hypocotyl–PR junction of *lax3/aux1-21* seedlings, only anomalous divisions in the outermost layers of the proliferated pericycle bundle occurred, whereas all AR stages were present under IBA + Kin treatment, even though they were characterized by anomalous cell divisions (Fig. 5H, I, arrows). As in IBA + Kin-treated *aux1-21* seedlings, early AR stages were prevalent, and, as in *lax3*, the rare protruding ARs showed a multilayered root cap. Moreover, as in the single mutants, the AR radial pattern was regular, as was the acropetal differentiation of protoxylem and its exarch position.

### SCR and AUX1 are expressed in the AR meristemoid founder cells in TCLs, and AUX1 is also expressed in cells leading to xylogenesis

**Fig. 6.**
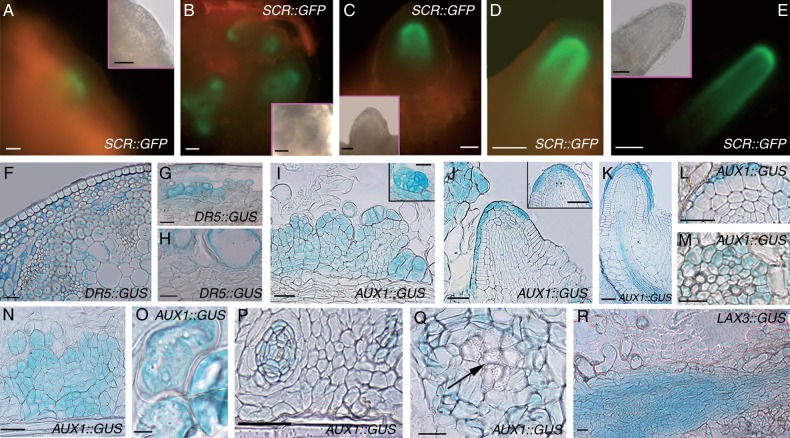
Expression of *SCR* (A–E), *AUX1* (I–Q) and *LAX3* (R) during AR formation and xylogenesis in TCLs, and endogenous auxin presence before and during TCL culture (F–H), by the use of *SCR::GFP*, *AUX1::GUS*, *LAX3::GUS* and *DR5::GUS* lines. (A) Weak *SCR* expression in a superficial meristematic clump. (B) High *SCR* expression in the root meristemoids. (C–E) *SCR* signal localization in a stage VII AR (C), ARP (D) and mature AR apex (E). (A–E, microscopic fluorescence pictures. Insets in A–C and E are corresponding bright-field images). (F) Pattern of *DR5::GUS* in the basal inflorescence stem before culture. (G, H) Auxin accumulation in forming (G) and maturing (H) pitted cells. (I) *AUX1* expression in meristematic clumps and root meristemoids (inset). (J) Protruding ARP with high *AUX1* expression in the root cap, and weak expression in the QC (asterisks in the inset). (K) Protruded AR with strong *AUX1* expression in the lateral cap, and forming epidermis and vasculature. (L, M) *AUX1* expression in a few epidermal cells (L), and pericycle, protoxylem and protophloem poles (M) in the AR primary structure. (N–Q) *AUX1* expression in callus cells (N), in scattered cells with degenerating protoplast and lignifying cell wall (O), and in forming xylary nodules (P), and disappearance of expression in mature xylary elements (arrow) (Q). (R) *LAX3* expression in a vascular strand. Longitudinal sections (A–E, G–K, N–R) and cross-sections (F, L, M). Scale bars = 10 µm (M, O), 30 µm (F, H, R), 50 µm (A–E, I–L, N, P, Q and insets in A, C, E, I, J) and 100 µm (G and inset in B).

*SCR* expression was regularly present in the stem endodermis of the basal inflorescence of *SCR::GFP* plants, but disappeared in the endodermal derivatives in *SCR::GFP* TCLs. Expression reappeared in the meristematic clumps leading to AR meristemoids, and continued to be present in the latter (Fig. 6A, B). In stage VII domes, protruding ARPs and elongating ARs, *SCR* expression was localized in the same AR stages from intact hypocotyls (Fig. 6C–E; compare with Fig. 2C–E).

In the basal stem inflorescence internodes of *DR5::GUS* plants, auxin was detected in randomly located cortical and endodermal cells (Fig. 6F). In the TCLs excised from these internodes and cultured with IBA + Kin, auxin accumulated in root meristemoids and ARP tips, but also in the QC, inner columella and differentiating protoxylem of the mature ARs, confirming previous results (Della Rovere *et al*., 2013). In addition, the cells involved in xylogenesis showed auxin accumulation (Fig. 6G, H).

*AUX1* was precociously expressed in cultured *AUX1::GUS* TCLs, marking the meristematic clumps and the AR meristemoids (Fig. 6I and inset). Expression continued in ARPs, i.e. forming cap, epidermis and, faintly, QC and surrounding initials (Fig. 6J and inset), and in protruding ARs, i.e. the lateral cap, and forming epidermis and vasculature (Fig. 6K). In the primary structure of the elongated ARs, *AUX1* was expressed in some epidermal cells, pericycle and protophloem and protoxylem poles (Fig. 6L, M). *AUX1* expression was also present in groups of callus cells arising from proliferated inner endodermis (Fig. 6N), and in differentiating vascular strands and nodules originating from them, as in single/small groups of cells directly developing into pitted elements (Fig. 6O, P), with expression ceasing after the completion of cell wall lignification and developmental cell death (Fig. 6Q). Also *LAX3* expression occurred in all the xylogenic cell types, e.g. the vascular strands (Fig. 6R). Moreover, it occurred in the AR meristemoids, and ARPs/ARs as in intact hypocotyls under the same hormonal condition (Della Rovere *et al*., 2013, and data not shown).

### SCR-1, SHR-1 and AUX1 are necessary for AR formation in TCLs

**Fig. 7.**
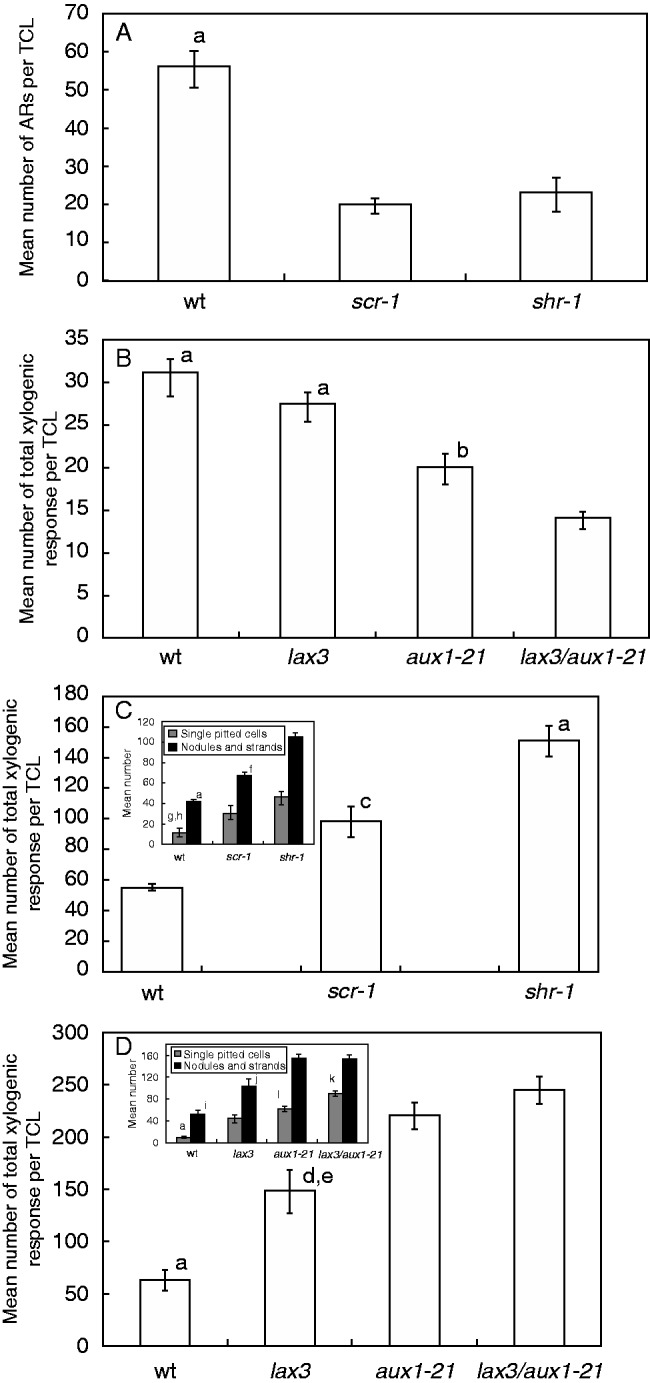
Macroscopic AR response (A, B) and xylogenic response (C, D) in TCLs from Ws (wt), *scr-1* and *shr-1* (A, C), and Col (wt), *lax3*, *aux1-21* and *lax3/aux1-21* (B, D) after 20 d of culture with IBA + Kin under darkness. (A, B) Mean number/TCL (±s.e.) of protruded ARs plus ARPs dissected from the callus under the stereomicroscope. (C, D) Mean number/TCL (± s.e.) of total xylogenic response, i.e. single cells with pitted cell walls, and xylogenic nodules and strands, counted together, in Ws (wt), *scr-1* and *shr-1* (C), and Col (wt), *lax3*, *aux1-21* and *lax3/aux1-21* (D). Insets in C and D show the mean number/TCL (± s.e.) of single pitted cells, and of nodules and strands*.* ^a^*P* < 0·001 with the other genotypes. ^b^*P* < 0·05 with *lax3/aux1-21*. ^c^*P* < 0·01 with the wt. ^d^*P* < 0·01 with *aux1-21*. ^e^*P* < 0·001 with *lax3/aux1-21.* ^f,g^*P* < 0·001 with *shr-1* within the same category. ^h^*P* < 0·05 with *scr-1* within the same category. ^i^*P* < 0·001 with *aux1-21* and *lax3/aux1-21* within the same category. ^j^*P* < 0·01 with the wt, *aux1-21* and *lax3/aux1-21* within the same category. ^k^*P* < 0·001 with *aux1-21* and *lax3* within the same category. ^l^*P* < 0·05 with *lax3* within the same category. Columns with no letter/the same letter are not significantly different (one-way ANOVA followed by Tukey’s post-test). *n* = 40 (A, B), *n* = 20 (C, D). Second replicate of the second year.

The basal stem internodes of *scr-1* and *shr-1* showed three irregular cortical layers between the epidermis and fibres, and no endodermis, as in the apical stem internodes (Fukaki *et al*., 1998). At the end of the culture period, macroscopic ARs only sporadically appeared on the basal internode TCLs of both mutants. Moreover, the mean number of ARs per rooting TCL was about 3-fold lower than in the wt, without significant differences between the two (Fig. 7A).

The basal stem internodes of *lax3*, *aux1-21* and *lax3/aux1-21* mutants showed the same regular structure as the wt internodes. At the end of the culture period, the mean number of ARs per rooting TCL was significantly reduced on *aux1-21* and *lax3/aux1-21* TCLs in comparison with the wt and *lax3*, whereas there was no significant difference between the latter two (Fig. 7B).

### AR formation is competitively inhibited by xylogenesis in *shr-1* and *scr-1* TCLs

**Fig. 8.**
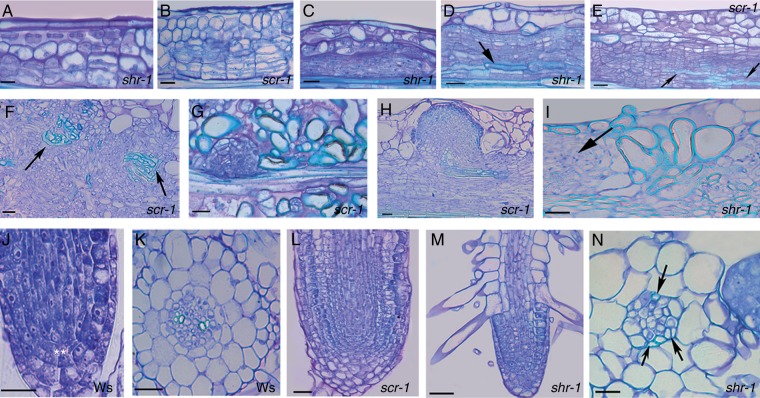
AR formation and xylogenesis in *shr-1* and *scr-1* TCLs during culture with IBA + Kin under darkness. (A, B) Early periclinal divisions in the innermost irregular cortical layer in *shr-1* (A) and *scr-1* (B) (day 3). (C) Bundle of meristematic cell files formed by the innermost irregular cortical layer in *shr-1*. (D, E) Xylary elements in the inner part of the meristematic bundle in *shr-1* (D) and *scr-1* (E) (day 7). The arrows show elements with pitted cell wall thickenings. (F) Details of xylogenic nodules (arrows) in a *scr-1* TCL. (G) Domed root meristemoid in *scr-1* TCLs, surrounded by lignified callus cells (day 7). (H) ARP not yet protruded from the explant in *scr-1*. (I) Callus cells highly expanded and with pitted cell walls around a late-formed meristemoid (arrow) in *shr-1* (day 14). (J, K) Regular AR tip, with the QC marked by the asterisks (J) and regular diarch primary structure (K) of ARs from wt (Ws) TCLs (day 20). (L, M) AR tips with anomalous stem cell niche and root cap cell enrichment in *scr-1* (L) and *shr-1* (M), and precocious differentiation of the epidermis with rhizoblasts in the latter (day 20). (N) Triarch AR from *shr-1* (xylem poles shown by the arrows). Longitudinal (A–J) and transverse (K, N) sections stained with toluidine blue. Scale bars = 20 µm (A, N), 30 µm (J, K) and 40 µm (B–I, L, M).

In both *shr-1-* and *scr-1* TCLs, cell groups in the innermost mutant cortical layer (i.e. that positioned as the endodermis in the wt) divided periclinally (Fig. 8A, B) at the same time (day 3) as the endodermis in wt TCLs. In contrast to the scattered groups generated by the wt endodermis, *shr-1* and *scr-1* periclinal derivatives formed a homogeneous meristematic bundle of superimposed cell files all along the explant (Fig. 8C). Strands of xylary elements with spiral/annular thickenings, but mainly pitted thickenings, appeared conspicuously within the meristematic bundle (Fig. 8D, E, arrows), whereas meristematic clumps, leading to AR meristemoids, appeared sporadically at its surface. In contrast, xylogenesis was reduced, but root meristemoid formation was enhanced, in the endodermis derivatives of wt TCLs. At day 7, the first xylogenic nodules and AR domes, surrounded by cells with pitted cell walls, appeared in *shr-1* and *scr-1* TCLs (Fig. 8F, G). In contrast, xylogenesis remained confined to the innermost part of the proliferating endodermis in wt TCLs, and the AR domes were not surrounded by lignified cells. During the second week, ARPs grew toward the epidermis in wt TCLs, and occasionally also in *shr-1*/*scr-1* TCLs (Fig. 8H); however, in the mutants, in contrast to the wt, the late-forming root meristemoids remained entrapped by lignified cells (Fig. 8I, arrow). At the end of the culture period, as in intact hypocotyls under the same treatment, and in contrast to the regular apical structure and the diarch xylem pattern of the ARs in wt TCLs (Fig. 8J, K), *shr-1* and *scr-1* ARs showed an anomalous stem cell niche and root cap, and no QC and endodermis. However, they showed precocious epidermis differentiation (Fig. 8L, M) and xylem overproduction leading to triarch/polyarch ARs (Fig. 8N). Moreover, xylogenesis was quantified as single pitted elements, nodules and strands. The total xylogenic response in the TCLs of both mutants was significantly higher than in wt TCLs, and significantly higher in *shr-1* TCLs than in *scr-1* TCLs (Fig. 7C); however, in both mutants, the main typology of the xylogenic response was made up of cells with pitted thickenings, both singly (Fig. 7C, inset) and located within nodules and strands (Fig. 8D, E, I).

### Xylogenesis is also enhanced in *aux1-21* and *lax3/aux1-21* TCLs concomitantly with AR reduction

**Fig. 9.**
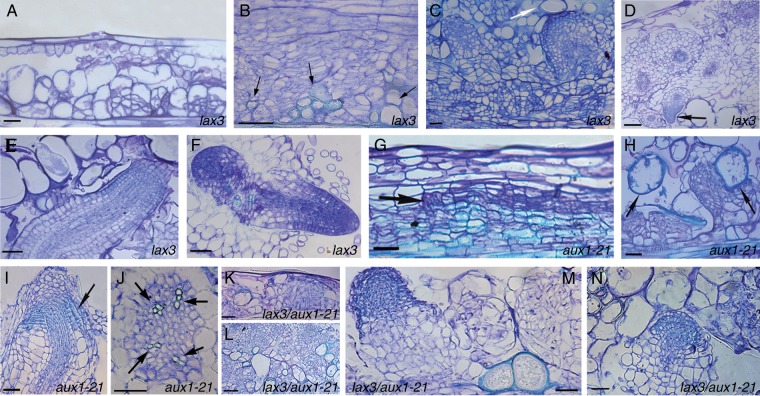
AR formation and xylogenesis in *lax3* (A–F), *aux1-21* (G–J) and *lax3/aux1-21* (K–N) TCLs during culture with IBA + Kin under darkness. (A) Early formation of scattered endodermis derivatives in *lax3* TCLs (day 3). (B) Cells with pitted thickenings (arrows) in the callus of a *lax3* TCL. (C) Anomalous *lax3* ARPs surmounted by pitted elements (arrow) (day 14). (D, E) Mature ARs within a day 20 *lax3* explant showing LRPs (arrow in D), a flattened apex and a multilayered root cap (E). (F) Primary region of a *lax3* AR showing two LRPs. (G) Bundle of endodermis periclinal derivatives in an *aux1-21* TCL with a xylogenic strand enriched in pitted elements (arrowheads) and a root clump in the outermost bundle layer (arrow) (day 7). (H) Meristemoids and domed ARPs confined with xylogenic cells (arrows) in *aux1-21* (day 14). (I) AR apex with early differentiation/expansion in the cortex, and meristematization in the epidermis and lateral cap (arrow) (*aux1-21* TCL, day 20). (J) Tetrarch xylem (arrows) within the stele of an *aux1-21* AR. (K) Formation of the bundle of endodermis derivatives in a *lax3/aux1-21* TCL (day 3). (L) Xylogenesis, mainly formed by pitted cells, in the callus of an *aux1-21* TCL (day 14). (M) A *lax3/aux1-21* ARP with anomalous structure near xylogenesis (day 14). (N) LRP on a *lax3/aux1-21* AR, with an excess of meristematization (day 20). Longitudinal (A–E, G–I, K–M) and transverse (F, J, N) sections stained with toluidine blue. Scale bars = 50 µm (A–C, E–K, M, N) and 100 µm (D, L).

The first divisions in the stem endodermis of *lax3* TCLs occurred as early as in wt TCLs, and similarly originated groups of derivative cells (Fig. 9A), forming scattered cell groups where vascular strands, nodules and single/small groups of pitted cells (Fig. 9B, arrows) differentiated. Moreover, as in wt TCLs, a lot of AR meristemoids were formed; however, *lax3* AR meristemoids developed into ARPs with a disorganized niche, numerous root cap initials and no QC. These ARPs were impeded in terms of protrusion by cells with pitted thickenings (Fig. 9C, arrow); nonetheless they became ARs, with most remaining entrapped within the explant (Fig. 9D). These ARs exhibited a flattened, cell-enriched, apex with a multilayered root cap (Fig. 9E), as in the intact hypocotyls under the same hormonal treatment, and formed a lot of lateral root primordia (LRPs) (Fig. 9D, arrow, and F). The few elongated ARs on *lax3* TCLs were frequently triarch/polyarch.

The quantification of xylogenesis at the end of the culture period revealed that the total response of *lax3* TCLs was significantly higher than in the wt explants (Fig. 7D), with pitted cells prevalent, both as single elements and within the strands/nodules (Figs 7D inset, and 9B).

In contrast to *lax3* TCLs, *aux1* TCLs exhibited a uniform bundle of endodermis periclinal derivatives along the explant, and an enrichment in meristematic cells and vascular strands, resembling *shr-1* and *scr-1* TCL responses (Fig. 9G; compare with Fig. 8D, E). AR clumps/meristemoids were randomly formed by the outermost layers of the bundle (Fig. 9G, arrow), and most remained entrapped within the pitted cells (Fig. 9H, arrows). The few meristemoids continuing development produced ARPs with early expanding cortex, but with a meristematic epidermis and lateral cap (Fig. 9I, arrow). The elongated *aux1-21* ARs were usually tetrarch (Fig. 9J). The quantification of the total xylogenic response showed that *aux1-21* TCLs produced significantly more xylary cells than *lax3* TCLs, and pitted cells in particular (Fig. 7D inset; Fig. 9G, arrowheads).

The bundle of endodermis derivatives formed in *lax3/aux1-21* TCLs was scattered as in *lax3* TCLs, but enriched in meristematic cells as in *aux1-21* TCLs (Fig. 9K; compare with A). Xylogenesis was enhanced as in *aux1-21* TCLs (Figs 7D and 9L), and similarly irregular domed ARPs appeared sporadically (Fig. 9M), and developed into ARs with the same anomalies as *aux1-21* ARs, but able to form LRPs as *lax3* ARs (Fig. 9N).

## DISCUSSION

The results show that SHR, SCR and AUX1 are needed for AR formation in intact hypocotyls and cultured TCLs, and the reduction in AR formation in *shr-1*, *scr-1*, *aux1-21* and *lax3/aux1-21* mutants is coupled to a promotion of xylogenesis.

### SHR and SCR mark AR progenitor cells, with AUX1 exhibiting a pivotal role

We demonstrated that *SCR* expression started in the AR founder cells in both intact hypocotyls and cultured TCLs, suggesting that SCR affected the initiation of ARs in the initiating tissues. *SCR* is activated by SHR in the PR (Helariutta *et al*., 2000), and the formation of a complex between the two proteins follows, and activates numerous downstream genes, including cell cycle components (Sozzani *et al*., 2010). It is possible that *SCR* is also activated by SHR during AR initiation, and that the transcription factors together activate downstream genes necessary for AR construction. Thus, the two genes might be necessary for the priming of the cell identity progenitors of the AR formation process, independently of the starting tissue (hypocotyl pericycle vs. stem endodermis), as is supported by the finding of similar, and strongly reduced, AR response in the hypocotyls and TCLs of their mutants. The same transcription factors were also necessary later in AR development, because QC definition and primary radial patterning depended on their action. In fact, *SCR* expression occurred at stage VII, when the QC is defined in the ARP (Della Rovere *et al*., 2013). In addition, the QC was not defined, and the endodermis was lacking, in the ARs of *scr-1* and *shr-1* TCLs and seedlings, as in their PR (Di Laurenzio *et al*., 1996; Helariutta *et al*., 2000).

Endogenous indole acetic acid (IAA) accumulation is an early event in LR formation (Benková *et al*., 2003). The present results show that it is also important in AR formation, because IAA accumulation in the pericycle at the hypocotyl–PR junction and in the basal inflorescence stem occurred before the first AR-forming divisions, and increased at their appearance in both intact hypocotyls and TCLs. In the PR tip, the cortex/endodermis initial cells need the presence of SHR and SCR, and of a local auxin increase, for the induction of downstream genes (Cruz-Ramírez *et al*., 2012), but a high level of *AUX1* transcription also occurs (Sozzani *et al*., 2010). We observed that the increase in endogenous IAA accumulation at AR stage I in the hypocotyl, and in the AR meristematic clumps in TCLs, was concomitant with the appearance of *AUX1* expression. This suggests that AUX1, mediating the auxin influx specifically in the AR-initiating cells, might be related to the priming activity of SHR and SCR. In contrast, *LAX3* expression occurred only from AR stage II onwards, suggesting that LAX3 was involved later than AUX1 in the AR formation process. In agreement with this, AR formation of *aux1-21* and *lax3/aux1-21* seedlings and TCLs was strongly inhibited, as in *shr-1* and *scr-1*, whereas that of *lax3* was comparable with that of the wt. It has been suggested that AUX1 recognizes endogenous IAA and not IBA, whereas IBA may be a substrate of LAX3 (Liu *et al*., 2012). In agreement with this, AUX1 expression was not enhanced by IBA + Kin, in contrast to LAX3 (Della Rovere *et al*., 2013; this study). It is thus possible that AUX1, and not LAX3, is involved in creating the initial endogenous IAA distribution essential for AR initiation by SHR and SCR. In contrast, the role of LAX3 is restricted to AR development, being necessary only from stage II onwards.

### There is an inverse relationship between AR formation and xylogenesis, governed by the same genes

Arabidopsis TCLs underwent xylogenesis in addition to AR formation under the exogenous IBA + Kin treatment (Falasca *et al*., 2004; this study), and the same treatment was necessary to cause ectopic metaxylem formation in the wt hypocotyls, even if this xylogenic response was limited in comparison with AR formation. Interestingly, xylogenesis increased in the seedlings and TCLs of all mutants, and this increase was always associated with increased xylem formation in the stele of the ARs, demonstrating that the two xylary processes were strictly related, sharing SHR, SCR, AUX1 and LAX3 activities.

Moreover, in seedlings and TCLs, xylogenesis began, and continued, where endogenous IAA accumulated, showing that high levels of IAA were essential to the process. We observed that ectopic metaxylem was the only xylogenic component in the intact hypocotyls, and ectopic metaxylem-like cells were the predominant component in TCLs. Metaxylem specification is known to be promoted by high auxin and high expression of the ZD-ZIP III genes (Ursache *et al*., 2014), and the HD-ZIP III transcription factor ATHB-8 is induced by auxin and involved in xylem specification *in planta*, and in xylogenesis in TCLs (Baima *et al*., 2000, 2014). ATHB-8, like other members of the family, is regulated by microRNA 165/6 produced by the SHR–SCR complex (Carlsbecker *et al*., 2010). Moreover, *shr-1* and *scr-1* seedlings are known to exhibit a high endogenous auxin content (Lucas *et al*., 2011; Moubayidin *et al*., 2013), and no formation of microRNA 165/6 (Muraro *et al*., 2014). Thus, a high endogenous auxin level and the absence of degradation of HD-ZIP III transcription factors might explain the observed enhancement of xylogenesis in the seedlings and TCLs of these mutants, and support the importance of SHR and SCR also in the xylogenic process. The exclusive ectopic metaxylem which occurs in the hypocotyl of *lax3*, *aux1-21* and their double mutant, and the prevalent ectopic metaxylem-like production of their TCLs, might be indicative of an endogenous IAA accumulation also in the latter mutants. Xylogenesis was enhanced in hypocotyls and TCLs of *aux1-21* and *lax3/aux1-21* in comparison with *lax3*, and this event was concomitant with a highly reduced rooting in the *aux1* mutants only. The non-utilization of the endogenous auxin in the AR process might have caused an excess of endogenous auxin in the *aux1* mutants, enhancing xylogenesis as an alternative to rooting. Consistent with this, high xylogenesis is caused by IAA in walnut microcuttings recalcitrant to rooting (Reverberi *et al*., 2001).

Thus, there is an inverse relationship between AR formation and xylogenesis, but with an important difference between the auxin influx carriers, highlighted by the response of their mutant TCLs. In fact, in *lax3* TCLs, ARs were formed, but xylogenesis trapped them within the explant, blocking only their macroscopic protrusion. In contrast, in the TCLs of *aux1-21* single/double mutants, the trapping effect by the xylogenic cells was limited, because the bulk of the ARs were arrested at early stages, whereas xylogenesis was overproduced. This means that xylogenesis occurred as an alternative programme to AR formation in *aux1-21* single/double mutant TCLs, from the first cell divisions onwards. Thus, in contrast to LAX3, AUX1 might exert a pivotal role in rhizogenesis/xylogenesis switching, in accordance with its role in other developmental processes, including *in vitro* organogenesis (Kakani *et al*., 2009; Péret *et al*., 2012).

In conclusion, AR formation and xylogenesis are developmental programmes which are inversely related, but involve a fine-tuning by the same proteins, i.e. SHR, SCR and AUX1. The pericycle activity is central for the equilibrium between xylary development and AR formation in the hypocotyl, with a role for AUX1 in the switch between the programmes, necessary for their balance. The results also open the way to understanding the genetic basis of AR recalcitrance in *in vitro* cultured cuttings in which block of AR formation is associated with enhanced xylogenesis resulting in unsuccessful micropropagation.
